# Monoclonal Antibody Therapies against Anthrax

**DOI:** 10.3390/toxins3081004

**Published:** 2011-08-15

**Authors:** Zhaochun Chen, Mahtab Moayeri, Robert Purcell

**Affiliations:** 1 Laboratory of Infectious Diseases National Institute of Allergy and Infectious Diseases, National Institutes of Health, Bethesda, MD 20892, USA; Email: rpurcell@niaid.nih.gov; 2 Laboratory of Bacterial Diseases, National Institute of Allergy and Infectious Diseases, National Institutes of Health, Bethesda, MD 20892, USA; Email: mmoayeri@niaid.nih.gov

**Keywords:** *Bacillus anthracis*, anti-PA mAbs, anti-LF mAbs, anti-EF mAbs, anti-capsule mAbs, post-exposure treatment of anthrax, a cocktail of mAbs

## Abstract

Anthrax is a highly lethal infectious disease caused by the spore-forming bacterium *Bacillus anthracis*. It not only causes natural infection in humans but also poses a great threat as an emerging bioterror agent. The lethality of anthrax is primarily attributed to the two major virulence factors: toxins and capsule. An extensive effort has been made to generate therapeutically useful monoclonal antibodies to each of the virulence components: protective antigen (PA), lethal factor (LF) and edema factor (EF), and the capsule of *B*.* anthracis*. This review summarizes the current status of anti-anthrax mAb development and argues for the potential therapeutic advantage of a cocktail of mAbs that recognize different epitopes or different virulence factors.

## 1. Anthrax Disease and Its Virulence Determinants

*Bacillus anthracis*, the causative agent of anthrax, is a Gram-positive, spore-forming bacterium that infects mostly herbivores. Humans are occasionally infected when exposed to contaminated animal products. However, anthrax poses a great threat as an emerging bioterror agent, highlighted by the anthrax attacks in 2001 [1,2].

There are three forms of anthrax disease, cutaneous, gastrointestinal and inhalational anthrax, depending on the route of infection. Inhalational anthrax is the deadliest form and the form used as a biological weapon in 2001. The high lethality of inhalational anthrax is largely attributed to the efficient replication of the bacterium and the action of its toxins. Following inhalation, spores are taken up either by alveolar macrophages or pulmonary dendritic cells and transported to local lymph nodes. These spores then germinate over the course of 2 to 43 days. Clinical symptoms develop rapidly after germination, and coincide with the production of toxins. Actively dividing bacilli produce three toxin components: protective antigen (PA), lethal factor (LF) and edema factor (EF). PA binds to cellular receptors and acts as a vehicle to deliver LF or EF into the cytosol where they exert their enzymatic activities (for review, see van der Goot, G. & Young, J.A. 2009) [3]. LF is a zinc-dependent protease that cleaves mitogen-activated protein kinase kinases [4,5]. EF is a calcium-calmodulin-dependent adenylate cyclase [6]. The combination of PA with LF results in lethal toxin (LT). LT can replicate symptoms of anthrax disease when injected into animals (for review, see Moayeri, M. & Leppla, S.H. 2009) [7]. PA combines with EF to form edema toxin (ET) which can produce a range of toxic effects in the host (for review, see Moayeri, M. & Leppla, S.H. 2009) [7]. 

In addition to the anthrax toxins (LT and ET), the vegetative bacillus also produces a capsule composed of a polymer of D-glutamic acid linked by γ-peptidyl bonds (γ DPGA). The toxins and the capsule comprise two major virulence factors that are associated with anthrax pathogenesis. The anthrax toxins play a key role in virulence by suppressing immune cell and cytokine responses, thereby promoting bacterial survival at early stages of infection, while inducing the shock-like death associated with anthrax at later stages following bacterial outgrowth in the blood [8]. On the other hand, the γ DPGA capsule allows bacteria to evade phagocytosis and has been shown to be essential for bacterial dissemination in the mouse and primate animal models [9,10]. The genes responsible for producing these two virulence factors are carried by two plasmids, pXO1 and pXO2, respectively. The loss of either plasmid results in attenuation of virulence [11,12], confirming the essential role of each factor for full virulence. 

## 2. The Limitations of Currently Recommended Post-Exposure Treatment

Current CDC recommendations following potential exposure to aerosolized *B. anthracis* spores are 60 days of oral antibiotics combined with a 3-dose series of the PA-based anthrax vaccine (anthrax vaccine adsorbed, AVA, BioThrax) [13]. While antibiotics and vaccination are an integral part of medical care, both have limitations. Antibiotics are effective in killing bacteria, but they are unable to clear released toxins from the bloodstream. Thus, unless exposure is diagnosed early enough for antibiotic treatment to prevent significant bacterial replication, patients will succumb to toxin-induced disease even after the killing of all bacteria [1]. In addition, there is growing concern about the possibility that a future bioterror attack could involve antibiotic resistant strains. Mutant strains that are resistant to the currently recommended antibiotics doxycycline and ciprofloxacin are rare in nature, but such resistant strains could be readily generated using straightforward experimental procedures in the laboratory [14,15]. The need for up to 60 days of antibiotic therapy tends to decrease compliance, as seen in the treatment associated with the 2001 attacks, for which the adherence rate was estimated to be 42% [16,17]. The current PA-based vaccine requires repeated administration and at least 4 weeks for development of anti-PA protective titers. Given the short incubation time and rapid disease progression of inhalational anthrax, vaccination is unlikely to afford protection after exposure. Thus, there exists a need for improved therapies to augment available treatment options for inhalational anthrax.

## 3. Passive Immunization through Treatment with mAbs

Passive immunization with protective antibodies represents an attractive option to augment the current post-exposure treatment of anthrax since it can provide immediate and extensive protection that is not dependent on the host response. Indeed, passive immunization with protective antibody has been considered to be the only available countermeasure in biodefense [18]. The overwhelming evidence indicates that antibodies are key players in conferring immunity to anthrax [19,20,21,22]. Thus, during the past 10 years, extensive research has been focused on development of therapeutic antibodies to target anthrax. This review summarizes the current status of therapeutic mAbs directed against the major virulence factors: PA, LF, EF and capsule. Furthermore, an argument for the possible therapeutic advantage of a cocktail of several mAbs that recognize different epitopes or different virulence factors (PA, LF, EF and capsule) is presented. 

### 3.1. Current Status of Anti-Anthrax mAb Development

#### 3.1.1. Anti-PA mAbs

The central role of PA in the pathophysiology of anthrax makes it an excellent therapeutic target. Vaccination with the PA-based human anthrax vaccine [23] or purified PA [24,25,26] results in the generation of a protective immune response. Passive immunization with polyclonal antibodies against PA is highly protective against challenge with *B. anthracis* spores [27,28,29]. Moreover, antibody titers against PA correlate with protective immunity against spore challenge [19,20,21,22]. The human polyclonal antibodies (anthrax immune globulin, AIG) from plasma of human volunteers who have been vaccinated with AVA have been recommended for use as an Emergency Investigational New Drug. The recent treatment with AIG of a patient who naturally acquired inhalation anthrax showed beneficial effect [30]. However, mAbs are the preferred choice for immunoprophylaxas as they offer several advantages over polyclonal antibodies, including defined specificity, reproducible efficacy, unlimited supply, high purity and increased safety. So far, greater than ten highly potent anti-PA neutralizing mAbs have been generated using different approaches [31,32,33,34,35,36,37,38,39,40]. These antibodies neutralize PA by different mechanisms, which include (i) inhibition of receptor binding [35,36,37,40], (ii) interference with PA heptamer formation [41], (iii) interference with LF or EF binding to PA [31], (iv) blockage of the enzymatic cleavage of PA into PA63 [34], and (v) disruption of preformed PA heptamer through formation of a supercomplex [39,42]. Some of the mAbs are murine-derived and are not useful in clinical applications because they will elicit detrimental anti-antibody immune responses in humans unless “humanized”. With the advent of new antibody technologies, it is possible to generate fully human or human-like mAbs. Currently, six such clinically useful anti-PA mAbs are available (Table 1) and each of them will be discussed below.

**Table 1:** Human and human-like anti-PA neutralizing monoclonal antibodies.

mAb	Origin	Epitope (Domain)	Neutralizing Mechanism	Animal Model for *in vivo* Protection	Reference
**Abthrax**	Human	IV	Inhibition of receptor binding	Rat, rabbit and monkey	[37]
**AVP-21D9**	Human	III	Interference with toxin assembly	Rat ^1^ and rabbit ^2^	[38,43]
**ETI-204**	Humanized	IV	Inhibition of receptor binding	Rabbit	[40]
**MDX 1303**	Human	III	Disruption of preformed PA heptamer	Rabbit and monkey ^3^	[39]
**IQNPA**	Human	IV	Inhibition of receptor binding	Mouse ^4^	[35]
**W1**	Chimpanzee	IV	Inhibition of receptor binding	Rat and mouse	[36]

^1^ Fischer 344 rats challenged with LT; ^2^ Rabbits challenged with virulent *B. anthracis* Ames spores; ^3^ Monkeys challenged with virulent *B. anthracis* Ames spores; ^4^ A/J mice challenged with toxigenic *B. anthracis* Sterne spores.

Abthrax (Raxibacumab) from Human Genome Sciences (HGS) is a fully human mAb derived from a human antibody phage display library licensed by HGS from Cambridge Antibody Technology [37]. The mAb presumably binds to domain IV of PA with an affinity of 2.78 nM and inhibits the binding of PA to its receptor. The *in vivo* protection was initially demonstrated in a rat toxin-challenge model and pre- and post exposure protection was further demonstrated in both New Zealand white rabbits and cynomolgus monkeys following lethal challenge of *B. anthracis* Ames spores. AVP-21D9 from Avanir Pharmaceuticals is a fully human mAb that was generated from human peripheral blood lymphocytes of AVA-immunized donors. The mAb has very high affinity with a *K*_d_ of 0.082 nM and inhibits PA heptamer formation [43,44]. The protective efficacy was initially demonstrated in a rat toxin-challenge model and pre- and postexposure protection was subsequently confirmed in Dutch-belted dwarf and New Zealand white rabbits following lethal challenge with virulent *B. anthracis* Ames spores [38]. ETI-204 (Anthim) from Elusys Therapeutics is a humanized, affinity-improved variant of mouse monoclonal antibody, 14B7 [31]. The mAb binds to domain IV of PA with an affinity of 0.33 nM and inhibits PA binding to receptor. Pre- and postexposure protection was demonstrated in New Zealand white rabbits following lethal challenge of *B. anthracis* Ames spores [40]. MDX1303 (Valortin) from PharmAthene/Medarex is a fully human mAb that was generated from HuMab transgenic mice that were engineered to express human immunoglobulin [45]. The mAb recognizes domain III of PA and potentially disrupts preformed PA heptamers by formation of a supercomplex in a manner similar to what was described for related antibody 1G3 [42]. The binding affinity has not been reported. Interestingly, the neutralizing activity of the mAb is dependent on Fc receptor. Pre-exposure protection was demonstrated in both New Zealand white rabbits and cynomolgus monkeys following lethal challenge with *B. anthracis* Ames spores [39]. IQNPA from IQ Corporation is a fully human mAb that was developed from peripheral blood lymphocytes from anthrax vaccine-immunized donors using electrofusion hybridoma technology. The mAb recognizes domain IV of PA and presumably inhibits the binding of PA to its cell receptor. Preexposure protection was determined in A/J mice challenged with a lethal dose of unencapsulated toxigenic Sterne strain [35]. W1 from the National Institute of Allergy and Infectious Diseases is a chimpanzee/human chimeric mAb that was recovered from chimpanzees immunized with PA by phage display library technology. W1 has the highest affinity among neutralizing antibodies which interact with the receptor-binding domain IV of PA, with *K*_d_ of 0.04 nM. W1 demonstrated high protective potency in rats using toxin bolus and infusion challenge models as well as in A/J mice challenged with a lethal dose of *B. anthracis* Sterne spores [36,46].

Currently, HGS has completed safety studies of Abtrhax in humans [47] and was awarded a contract to provide a stockpile of 65,000 doses for treatment of inhalation anthrax. Elusys Therapeutics has completed a Phase 1 clinical study with ETI204 and the drug has received Fast-Track and Orphan Drug status by the FDA. Similarly, a Phase 1 clinical trial with mAb MDX1303 has been completed and the mAb has also received Fast-Track and Orphan Drug status by the FDA. Since different neutralizing mechanisms are used by these mAbs and different animal models, challenge doses, antibody doses, and routes of administration have been used in their testing, it is difficult to directly compare these anti-PA mAbs. Furthermore, limited resources and high costs of testing have delayed the testing of some mAbs in the preferred rabbit or monkey inhalational anthrax models. However, it has been shown that affinity correlates well with neutralizing activity; higher affinity conferred better protection for mAbs that are specific to domain IV of PA [36,48]. Since mAbs that are specific to domain IV of PA neutralize by inhibiting the binding of PA to its receptor, it is essential that mAbs bind to PA with higher affinity than the interaction between PA and its receptor. A range of affinities for PA and its receptors has been reported, from 0.17 to 33.3 nM [49,50,51,52]. To compete effectively with the PA receptor for PA binding, mAbs need to have an affinity greater than this range. Comparison among four mAbs that neutralize PA by inhibiting the binding of PA to its receptor indicates that the only mAb that truly falls outside the range of affinities for PA and its receptor is anti-PA W1 (Table 2). However, the efficacy of W1 relative to other antibodies has not been assessed in the rabbit or non-human primate models. 

**Table 2:** Comparison of human or human-like mAbs that recognize the same receptor-binding domain of PA.

mAb	Affinity (*K_d_*)	Antibody dose for 100% protection	Reference
**Abthrax**	2.78 nM	1.5 mg/kg in rat ^2^, 40 mg/kg in rabbit ^3^, 40 mg/kg in monkey ^4^	[37]
**ETI-204**	0.33 nM	4 mg/kg in rabbit ^3^	[40]
**IQNPA**	ND ^1^	7.2 mg/kg in mouse ^5^	[35]
**W1**	0.04 nM	0.021 mg/kg in rat ^2^, 1.6 mg/kg in mouse ^6^	[36]

^1^ ND: not determined; ^2^ Fischer 344 rats were challenged with LT; ^3^ New Zealand white rabbit inhalational anthrax model with *B. anthracis* Ames spores; ^4^ Cynomolgus macaque inhalational anthrax model challenged with *B. anthracis* Ames spores. 90% protection at the dose indicated; ^5^ A/J mice were challenged with 24 LD_50_ of *B. anthracis* Sterne spores; ^6^ Unpublished data. A/J mice were challenged with 2 × 10^7^ Stern spores (~1000 LD_50_). All PBS-treated mice died 48 h after challenge.

#### 3.1.2. Anti-LF mAbs

LF plays a pivotal role in cytotoxicity and progression of disease in the infected host [53]. Currently, several neutralizing mAbs specific to LF have been reported. Some of the well-characterized anti-LF mAbs are listed in Table 3. Most of them are murine-derived and are not suitable for use in humans, and therefore, will not be discussed further. IQNLF is a fully human mAb, while LF10E and LF11H are chimpanzee/human chimeric mAbs. IQNLF recognizes domain I of LF and thus likely inhibits the binding of LF to PA which occurs through this domain. A single dose of 180 µg of IQNLF conferred 100% protection to A/J mice that were challenged with 24 LD_50_ of *B. anthracis* Sterne spores [35]. MAbs LF10E and LF11H bind to domain I of LF with affinities of 0.69 nM and 7.4 nM, respectively. Interestingly, neither mAb inhibits the binding of LF to PA. Initial experiments showed that LF10E and LF11H at substoichiometric or equal molar ratios of 1:0.5 and 1:1 of LF to mAb, respectively, conferred 100% protection of Fischer 344 rats from challenge with LT [54]. A recent experiment showed that 200 µg of LF10E provided 100% protection of A/J mice challenged with 1000 LD_50_ of *B. anthracis* Sterne spores (data not shown). 

**Table 3:** Characteristics of available anti-LF neutralizing mAbs.

mAb	Origin	Affinity (*K*_d_)	Epitope (Domain)	*In vitro* Neutralization (EC_50_)	*In vivo* Neutralization	Reference
**LF8**	Mouse	ND ^1^	I	+ (ND) ^3^	Athymic nude mouse ^4^	[55]
**9A11**	Mouse	70.1 nM	ND	1.3 nM	Balb/C mouse ^5^	[33]
**10G3**	Mouse	20 nM ^2^	I	+ (ND)	Fischer 344 rat	[56]
**2E7**	Mouse	87 nM ^2^	I	+ (ND)	Fischer 344 rat	[56]
**3F6**	Mouse	40 nM ^2^	I	+ (ND)	Fischer 344 rat	[56]
**5B13B1**	Mouse	2.62 nM	III	1.4 nM	Fischer 344 rat	[57]
**3C16C3**	Mouse	8.18 nM	III	4.2 nM	Fischer 344 rat	[57]
**IQNLF**	Human	ND	I	0.1 nM	A/J mouse	[35]
**LF10E**	Chimpanzee	0.69 nM	I	0.1 nM	Fischer 344 rat and A/J mouse	[54]
**LF11H**	Chimpanzee	7.4 nM	I	400 nM	Fischer 344 rat	[54]

^1^ ND: not determined; ^2^ Calculated IgG concentration for 50% maximal binding in ELISA based on original data; ^3^ Positive in *in vitro* neutralization assay, but EC_50_ was not determined; ^4^ MAb and LT were injected intravenously into athymic nude (nu/nu) mice daily; ^5^ Balb/C mice were injected with mAb and then challenged with LT intraperitoneally.

#### 3.1.3. Anti-EF mAbs

Fewer neutralizing mAbs to EF have been reported as compared to mAbs against PA and LF (Table 4) [58,59,60]. This is perhaps because EF has been considered to contribute less to the lethality of anthrax infection [61,62] and epitopes in EF that elicit nonneutralizing mAbs appear to be immunodominant as most mAbs to EF reported thus far do not neutralize EF [58,59,60]. Nevertheless, one of the EF-neutralizing mAbs, EF13D is very promising for therapeutic use [59]. EF13D is a chimpanzee/human mAb that can neutralize EF *in vitro* in the subnanomolar range. The therapeutic usefulness of the antibody was demonstrated by its efficient prevention of local edema formation in a murine footpad model, as well as protection of mice from death following edema toxin challenge. EF13D binds to a conformational epitope within domain III of EF with very high affinity (*K*_d_ of 0.05–0.12 nM). The antibody can not only inhibit the binding of calmodulin (CaM) (which is required for activity) to EF, but also can displace pre-bound CaM from the EF-CaM complex.

**Table 4:** Characteristics of anti-EF neutralizing mAbs.

mAb	Origin	Affinity (*K*_d_) ^1^	*In Vitro* Neutralization ^2^	*In Vivo* Neutralization ^3^	Reference
**9F5**	Mouse	2 nM	Yes	ND	[58]
**1E6**	Mouse	5 nM	Yes	ND	[58]
**7G10**	Mouse	9 nM	Yes	ND	[58]
**9F3**	Mouse	830 nM	Yes	No	[60]
**EF13D**	Chimpanzee	0.05–0.12 nM	Yes	Yes	[59]

^1^ The affinities for mAbs 1E6, 7G10, 9F5 and 9F3 were estimated from binding assay by ELISA and affinity for mAb EF13D was determined by surface plasmon resonance on Biacore; ^2^ The *in vitro* neutralization activity was measured by the ability of antibody to inhibit cyclase activity of EF; ^3^* In vivo* neutralization assay was not determined for mAbs 1E6, 7G10 and 9F5 (ND). Prolonged but, ultimately no survival of A/J mice treated with mAb 9F3 was observed following challenge with Sterne spores. MAb EF13D prevented local edema formation and protected mice from death following challenge with ET.

#### 3.1.4. Anti-Capsule mAbs

The capsule is poorly immunogenic and acts as a thymus-independent, type 2 antigen [63]. Due to the lack of effective antibody response to the capsule, the antiphagocytic nature of the capsule ensures the unchecked proliferation of bacilli. Clearly, the current PA-based vaccine would not elicit the production of anti-capsule antibodies and therefore, such antibodies are absent from AIG currently stocked for use as an Emergency Investigational New Drug. The inherently weak immunogenicity of the capsule can be significantly enhanced through conjugation to a strong immunogenic protein carrier [64,65,66,67] or by administration of γDPGA in combination with a CD40 agonist mAb [68]. By these approaches, several murine and chimpanzee-derived mAbs that promote effective oposonophagocytosis of *B. anthracis* have been isolated (Table 5) [68,69,70]. Passive immunization with these specific anti-capsule mAbs conferred significant protection in naïve mice against spores of the Ames strain. In comparison, the chimpanzee-derived anti-capsule mAbs 11D and 4C had an order of magnitude higher binding affinities and conferred better protection than murine mAbs [70]. More importantly, 11D and 4C not only provided pre-exposure protection, but also protection against lethal infection when mAbs were administrated as late as 20 h after spore challenge. These mAbs could be particularly useful for treatment of infections with antibiotic-resistant strains.

**Table 5:** Comparison of some well characterized anti-capsule neutralizing mAbs.

mAb	Origin	Affinity (*K*_d_) ^1^	Antibody Dose for 100% Protection ^2^	Reference
**F26G3**	Mouse	370 nM	2–4 mg	[69]
**F24F**	Mouse	500 nM	2 mg	[69]
**F26G4**	Mouse	510 nM	2–4 mg	[69]
**4C**	Chimpanzee	36 nM	0.3 mg	[70]
**11D**	Chimpanzee	64 nM	0.3–1 mg	[70]

^1 ^The *K*_d _was determined by fluorescence tryptophan perturbation assay; ^2^ Murine model of pulmonary anthrax: Balb/c mice were challenged with lethal infection of Ames spores.

### 3.2. Maximizing the Efficacy of Antibody Therapy by Targeting Multiple Epitopes

Even though a single mAb that neutralizes one of the anthrax virulence components (PA, LF, EF and capsule), particularly PA, may be sufficient to confer significant protection, a mixture of more than one mAb against different targets or epitopes could maximize the protective efficacy. Such combinatorial therapy would not only broaden the spectrum of protection but may also synergize protective efficacy. The synergistic effect of multiple mAbs has been well documented in other diseases. A dramatic synergistic effect has been reported for mAbs to botulinum neurotoxin type A (BoNT/A) [71]. A mixture of three anti-BoNT/A mAbs increased protective efficacy by at least 1000-fold when compared with individual mAbs used alone. A similar effect has been observed for anti-tetanus mAbs, where a combination of two mAbs provided complete protection against a lethal tetanus toxin challenge in mice, while single mAbs were not protective [72]. The benefit of mAb combinations in the neutralization of Rift Valley fever, HIV and dengue viruses has also been reported [73,74,75,76]. 

For anthrax, several anti-PA mAbs that recognize different epitopes and thereby employ different neutralization mechanisms have been generated. Each mAb, regardless of differences in neutralizing mechanism, demonstrated highly potent neutralizing activity [31,32,33,34,35,36,37,38,39,40]. However, a point mutation in the neutralization epitope introduced naturally or intentionally can abolish antibody activity as demonstrated by the involvement of a single amino acid as crucial to neutralizing antibody function in the PA-antibody interaction [77]. Obviously, a cocktail of anti-PA mAbs that recognize different epitopes on PA would broaden the spectrum of protection, which would be much more difficult to overcome by escape mutants since multiple point mutations in different epitopes essential to toxin function would be necessary for escape. Furthermore, it is possible that such a combination of different anti-PA mAbs with different specificities would synergize the protective efficacy. 

Synergy between anti-PA and anti-LF antibodies has also been investigated. The efficacy of three mouse mAbs recognizing domain 2 and domain 4 of PA and the *N*-terminus of LF, were tested in a mouse Sterne spore-challenge model, in combination and alone [34]. A combination of 1 µg of each mAb resulted in full protection while individual mAbs at doses of 1 µg or 10 µg conferred 0–25% protection. Synergy between anti-PA and anti-LF was also observed in a rat LT-challenge model with anti-PA W1 and anti-LF LF11H [54]. These results are consistent with the notion that although PA plays the central role in protective immunity antibodies against LF and EF can also play an important role in protection [78,79,80]. 

**Figure 1:**
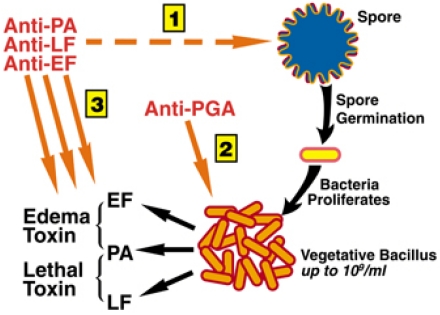
Comprehensive protection could be achieved by a combination of anti-PA, anti-LF, anti-EF and anti-PGA mAbs that target major steps of the infection process.

Anthrax is a complex disease involving several steps to establish infection, including spore germination, proliferation of bacilli and toxin production, leading to bacteremia and toxemia. Ideally, mAbs to each of the virulence factors could be used together, so that a comprehensive protection could be achieved by inhibiting multiple steps of infection (Figure 1). The finding that PA is present on the surface of the dormant spore and that antibodies to PA enhance spore phagocytosis and spore killing by macrophages *in vitro* [81,82,83] suggests that anti-PA mAbs may interfere with the early stage of infection. Furthermore, it has recently be shown that toxin function against macrophages and neutrophils is essential to avoiding bacterial clearance by these immune cells and establishing infection, confirming an important role for anti-toxin antibodies in early stages [53]. In addition to anti-toxin antibodies interfering with early steps in infection, the actively dividing vegetative bacteria can be killed by anti-capsule mAbs through opsonophagocytosis. High levels of toxin synthesized later in infection and responsible for lethality in this disease can also be neutralized by anti-PA, -LF and -EF mAbs. 

At the very least, the combination of anti-PA and anti-capsule mAbs may be sufficient for maximum efficacy since they combine both anti-bacterial and anti-toxin activities. Even though anti-PA mAb alone is very effective in protection, a greater therapeutic efficacy has been achieved by passive immunity from anti-PA mAbs in combination with antibiotics [41,84]. Thus, it is reasonable to assume that the same effect could be achieved by combination of anti-PA and anti-capsule mAbs. Actually, anti-capsule mAbs compare favorably with antibiotics in terms of treating antibiotic-resistance strains and providing a prolonged duration of protection. It is critically important that we are prepared to treat anthrax infection that involves antibiotic-resistant strains since such strains could be readily generated in the laboratory as described above. Anti-capsule mAb is a clear choice for treating such antibiotic-resistance strains. Human IgG1 has a considerably longer serum half-life than antibiotics (21 days *vs.* 0.1–0.2 day), and this extended duration of efficacy can be quite dramatic. For example, most antibiotics have to be taken once or more daily, but a single dose of an antibody may protect for more than 20 days. The duration of protection is especially important in anthrax treatment because anthrax spores can remain dormant in the lungs for an extended period of time [85,86] and a 60-day course of oral antibiotics is recommended, which has resulted in poor compliance [16,17]. By contrast, due to its longer half-life, 2–3 doses of mAbs may be sufficient to provide protection for more than 60 days. 

## 4. Conclusions

Several therapeutically useful anti-PA, anti-LF, anti-EF and anti-capsule mAbs have been generated. These mAbs used alone would most likely improve currently recommended post-exposure treatment of anthrax. Use of a cocktail of mAbs that target different epitopes or virulence factors could maximize the protective efficacy as it would not only broaden the spectrum of protection but may also synergize the protective efficacy. In particular, therapy that included an anti-capsule mAb could be useful for treatment of infections with antibiotic-resistant strains. 
